# Comprehensive assessment of the efficacy and safety of a clay mask in oily and acne skin

**DOI:** 10.1111/srt.13513

**Published:** 2023-11-05

**Authors:** Xianghua Zhang, Zhongxing Zhang, Han Tao, Xiaofeng He, Kungchi Hsu, Wenna Wang, Xiaofeng Fang, Andrew Steel

**Affiliations:** ^1^ L'Oréal Shanghai China; ^2^ Research and Innovation Center L'Oréal China Shanghai China

**Keywords:** acne, clay mask, oily skin, porphyrin counts, sebum control

## Abstract

**BACKGROUND:**

Oily skin, characterized by excessive sebum production, can lead to acne and have psychosocial impacts due to changes in appearance. Recent research has shown interest in treatments for oil control, with kaolin and bentonite emerging as promising options. Despite their potential, comprehensive studies on these ingredients are still in the nascent stages.

**AIM:**

This study aimed to assess the efficacy of a clay mask (La Roche‐Posay Effaclar Sebo‐Controlling Mask) in reducing skin oiliness and acne, and its safety for use.

**METHODS:**

In this study, 75 adults with oily or combination skin were enrolled and provided with a clay mask for twice‐weekly use over 4 weeks. Clinical assessments, using instruments like Sebumeter, Vapometer, and Corneometer, were conducted at baseline, and after 1, 2, and 4 weeks, evaluating acne lesions, skin irritation, sebum content, and skin hydration. Participant self‐assessment questionnaires were also utilized for subjective evaluation. Statistical analyses were performed accordingly.

**RESULTS:**

The study revealed significant improvements in acne‐related outcomes, sebum content, skin evenness, stratum corneum water content, and transepidermal water loss following the application of the clay mask. Pore area and porphyrin area showed no significant changes. Tolerance assessment showed reduced dryness and irritation, with self‐assessment indicating high product acceptability and perceived oil control effectiveness.

**CONCLUSION:**

This study demonstrated the clay mask's efficacy in managing acne and oily skin, improving hydration and texture. Significant improvements in skin parameters and high product safety were observed, supporting its suitability.

## 1 INTRODUCTION

Oily skin, characterized by an overproduction of sebum, is a widespread dermatological condition affecting a significant portion of the global population. Studies suggest that oily skin can lead to a shiny complexion, enlarged pores, and is often associated with acne due to the blockage of pores.^1^ These manifestations not only affect an individual's appearance but can also contribute to lowered self‐esteem and psychological distress.^2^ Despite the prevalence of oily skin, finding an effective treatment remains a substantial challenge in dermatology. Any treatment deemed successful must strike a balance between controlling excess oil production, maintaining skin hydration, and minimizing adverse effects. Moreover, environmental factors such as high temperatures can exacerbate sebum production, adding to the complexity of treating oily skin.^3^

Current treatments for oily skin, ranging from topical applications such as retinoids and salicylic acid to more advanced procedures like chemical peels and laser therapy, present their own set of challenges.^4^ Despite their efficacy, they are often associated with side effects like dryness and irritation and typically necessitate ongoing use to maintain results.^5^, ^6^

In light of the limitations of existing treatments, recent years have seen an uptick in research into less invasive treatments for oily skin. Notably, certain ingredients, including kaolin and bentonite, have shown promise in controlling oiliness without excessively drying the skin.^7^ Kaolin and bentonite are known for their strong oil‐absorbing and impurity‐extracting abilities, which may be particularly beneficial for severe cases of oily skin and acne.^8^ Additionally, many clay masks incorporate other oil‐controlling ingredients, such as activated charcoal, recognized for its high absorptive capacity and ability to draw out impurities from the skin.^9^ However, there is a lack of comprehensive studies examining the effects of these ingredients in combination over extended periods, making it important to understand their impact on the skin's oil production and overall health.

In this study, we aim to assess the efficacy and safety of a clay mask, enriched with cellulobeads, kaolin, bentonite, thermal spring water, and vitamin B5 (panthenol), improving the skin condition and acne in the management of oily skin, with great tolerance and safety.

## 2 METHODS

### 2.1 Study design and subjects

This study enrolled adults (70% women) aged 18–45 years old with either oily or combination, with at least 50% self‐declared sensitive skin (baseline sebum level ≥100 μg/cm^2^) recruited in Shanghai, China, in March 2023.

Inclusion Criteria: The study included participants who were aged between 18 and 45 years, had a skin type classified as either oily or combination, and self‐declared sensitive skin at least 50% of the time. Participants also had to have an open and closed comedone on the face or nose, with mild or moderate acne rated less than or equal to 2 on the ISGA scale. Furthermore, they had to be participants not currently participating in any other study involving the test area (face) during the research period, and those who had not participated in any similar study involving the test area (face) in the past month.

Exclusion criteria: Participants were excluded from the study if they had any chronic disease that could interfere with the test, such as asthma, insulin‐dependent diabetes, lupus, rheumatoid arthritis, or other immune/autoimmune diseases. The use of any prescription or over‐the‐counter medication applied to the skin of the test area (face), such as anti‐acne or hydrocortisone products, was also a disqualifying factor. Those who had been diagnosed with cancer within the past year, or had received cancer treatment within that period, or were currently taking preventive medication related to cancer were also excluded. Participants with any skin condition (such as eczema, psoriasis, rosacea), infection or injury in or around the test area (face), and those who are pregnant, planning to become pregnant during the study period, or currently nursing were also excluded from the study. All subjects provided signed informed consent before enrollment.

### 2.2 Intervention

Participants were given a clay mask for facial treatment. Initial use of the product was conducted at the research center, where participants were guided on proper application (let the clay mask stay for 5–10 min and then wash with water) and subsequently asked to complete a questionnaire. The mask was then distributed for home use, with instructions for twice‐weekly applications.

### 2.3 Clinical assessment

In a controlled environment, maintaining uniform temperature and humidity, a series of clinical assessments were performed on participants at baseline, and after 1, 2, and 4 weeks of the intervention. Prior to each assessment, participants acclimated to the environment for 30 min after washing their faces, excluding the forehead.

Dermatologists conducted a comprehensive evaluation of acne lesions, counting closed comedones (whiteheads), open comedones (blackheads), inflammatory papules, and pustules. Additionally, a tolerance test was conducted to assess the product's potential skin irritation and subjective discomfort effects. These effects included erythema, edema, dryness, scaling, and sensations of burning, stinging, itching, tightness, and tingling. Evaluations were carried out prior to product use and after each individual use.

### 2.4 Instrumental assessment

Sebum content was assessed using the Sebumeter SM‐815 (Courage & Khazaka, Germany). The device was applied once to the forehead, providing a reading that represented the sebum content of the skin in that area.

A Vapometer (Delfin, Finland) was employed to evaluate Transepidermal Water Loss (TEWL). Three separate measurements were taken and subsequently averaged to obtain a representative value of the skin's water loss rate.

Hydration level of the stratum corneum, the outermost layer of the skin, was determined using a Corneometer CM 825 (Courage + Khazaka, Germany). The device was used to take three separate measurements, the average of which provided the hydration level of the stratum corneum.

Frontal images of the face were captured using VISIA‐CR (Canfield, USA). These images were then processed using IPP software, which provided data regarding the proportion of pore area, skin color evenness, and the proportion of porphyrin area.

### 2.5 Self‐assessment

Subjective discomfort was assessed by having the participants rate their sensations of burning, stinging, itching, tightness, and tingling on a scale from 0 to 3. These scores corresponded to various degrees of discomfort, ranging from “unnoticeable” to “very noticeable,”.

Furthermore, participants completed questionnaires at the research center. These questionnaires were administered initially after the first use of the product at the center and subsequently after each assessment session (Immediate post‐treatment, Week 1, Week 2, and Week 4). The questionnaires provided additional insights into the participants' subjective experiences, perceptions, and satisfaction levels with the product.

### 2.6 Statistical analysis

Descriptive statistics, including count, mean, standard deviation, minimum, and maximum values, were generated for all measured values using Microsoft Excel. This provided an initial understanding of the data distribution and central tendencies. For comparison of metric data before and after product use, the Shapiro‐Wilk Test was conducted using SPSS software to assess the normality of the data change values. If the data followed a normal distribution, a paired t‐test was employed. If not, the Wilcoxon rank‐sum test for two related samples was utilized. The level of significance, α, was set at 0.05 for these tests. Ranking data were compared before and after product use employing the two related samples rank‐sum test. Comparisons between the test and control areas were conducted using a paired sample t‐test or rank‐sum test, depending on the nature of the data. Here, too, the level of significance, α, was set at 0.05.

## 3 RESULTS

### 3.1 Study flowchart

A total of 75 participants were screened, of whom 55 participants were intended for both clinical assessment and questionnaire feedback and 20 participants intended for questionnaire feedback only. This screening process led to the enrollment of 65 participants, comprising 45 participants for the clinical assessment/questionnaire group and 20 participants for the questionnaire‐only group. The study was successfully completed by 60 participants in total, with 40 participants from the clinical assessment/questionnaire group and 20 participants from the questionnaire‐only group, whose data were included for subsequent analysis.

### 3.2 Baseline patient characteristics

Mean ages of the participants were 38.13 ± 6.82 and 38.62 ± 6.58 years in the clinical assessment/questionnaire and questionnaire‐only groups, respectively; 68.30% of the participants were women, and 85.00% had self‐assessed sensitive skin. Lipid Index at baseline were 154.60 ± 32.18 and 157.25 ± 25.86 μg/cm^2^; ISGA Scores were 1.40 ± 0.50 and 1.30 ± 0.47; Sensitive skin self‐rating score were 5.70 ± 1.26 and 5.65 ± 0.99, respectively.

### 3.3 Treatment efficacy

In relation to acne‐related outcomes, both closed comedones and open comedones exhibited significant reductions immediately post‐treatment with improvement rates of 5.06% and 4.81% respectively. The reductions became more pronounced over the weeks, with rates of 25.75% and 25.38% at week 1, 32.18% and 47.12% at week 2, and 46.44% and 65.77% at week 4 (all *p* < 0.001).

There was no significant alteration in the stratum corneum water content immediately after treatment (*p* = 0.067). However, significant increases were observed at week 1 (14.33%, *p* < 0.001), week 2 (16.51%, *p* < 0.001), and week 4 (29.65%, *p* < 0.001), suggesting a cumulative hydrating effect of the product over time.

An immediate post‐treatment reduction of 3.09% in TEWL was recorded (*p* = 0.009), which signifies an enhancement in the skin barrier function. This reduction persisted and intensified over the weeks, with reductions of 11.00% at week 1, 16.32% at week 2, and 20.41% at week 4 (all *p* < 0.001).

Following the immediate post‐treatment, there was a marked reduction in skin oiliness by 68.97% (*p* < 0.001). This decline continued with rates of 24.26% at week 1, 24.22% at week 2, and 29.90% at week 4 (all *p* < 0.001).

**FIGURE 1:**
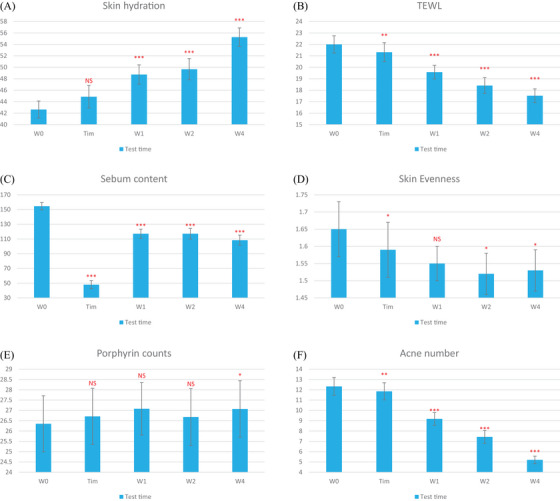
Changes from baseline in investigator global assessment (IGA) score of acne severity (A), Skin hydration (B), TEWL (C), Sebum content (D), Skin evenness (E), Porphyrin counts (F), Acne number.

**FIGURE 2:**
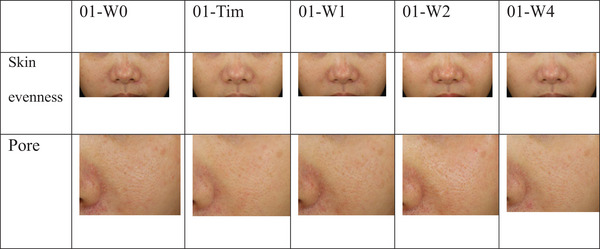
VISIA‐CR analysis illustration for an average case: Evaluation of skin evenness and pore characteristics from baseline (T0) to week 4 (W4).

An immediate improvement of 3.64% in skin evenness was noted post‐treatment (P = 0.030). Further improvements were observed at week 2 (7.88%, *p* = 0.021) and week 4 (7.27%, *p* = 0.018). Lastly, the proportion of the porphyrin area showed a slight increase over the 4‐week duration. This increase was not statistically significant (Figure 1, Figure 2).

### 3.4 Tolerance assessment

Objective assessment, including erythema, edema, dryness, and desquamation, showed a significant decrease in dryness and an overall reduction in total irritation from immediate post‐treatment to week 4. Subjective assessment, including burning, stinging, itching, and tightness sensations, revealed significant reductions in itching and tightness from immediate post‐treatment to week 4. The total discomfort score also significantly decreased during this period. The results are shown in Table 1 and Table 2.

**TABLE 1:** Result of Objective Irritation Parameters Assessment (60 Subjects).

	W0	Tim	W1	W2	W4
erythema	22 (0,0,2)	17 (0,0,2)	16 (0,0,1)	20 (0,0,1)	15 (0,0,1)
edema	1 (0,0,1)	1 (0,0,1)	0 (0,0,0)	0 (0,0,0)	0 (0,0,0)
dryness/scaling	37 (0,1,2)	16 (0,0,1)***	10 (0,0,1)***	12 (0,0,1)***	7 (0,0,1)***
desquamation	1 (0,0,1)	1 (0,0,1)	1 (0,0,1)	5 (0,0,1)*	0 (0,0,0)
Total Score	61 (0,1,3)	35 (0,0,3)***	27 (0,0,3)***	37 (0,0,3)**	22 (0,0,2)***

**TABLE 2:** Result of subjective discomfort parameters assessment (60 Subjects).

	W0	Tim	W1	W2	W4
burning	3 (0,0,1.5)	1 (0,0,1)	0 (0,0,0)	0 (0,0,0)	0 (0,0,0)
stinging	1 (0,0,0.5)	0 (0,0,0)	0 (0,0,0)	0 (0,0,0)	0 (0,0,0)
itching	13.5 (0,0,1.5)	1 (0,0,0.5)***	1.5 (0,0,0.5)***	0 (0,0,0)***	0.5 (0,0,0.5)***
tightness	47.5 (0,1,2)	15.5 (0,0,2)***	11.5 (0,0,1)***	5 (0,0,1)***	4.5 (0,0,1)***
tingling	0 (0,0,0)	0 (0,0,0)	0 (0,0,0)	0.5 (0,0,0.5)	0 (0,0,0)

### 3.5 Self‐assessment

Immediately after application, over 90% of participants found the product easy to apply and wash off, with 96.7% and 90% agreement respectively. Notably, 100% of the participants reported the product's oil control effectiveness after 1 week of use. This perceived benefit was consistently reported across week 2 and week 4, with 100% and 98.3% of participants, respectively. In terms of skin comfort, 91.7% of participants at week 1 and 95% at week 4 reported a lack of skin tightness or dryness. By the end of the study at week 4, other positive skin changes were reported by over 95% of participants, including reduced visibility of skin flaws, improved skin appearance, and reduced skin discomfort.

## 4 DISCUSSION

This study evaluated the efficacy, safety and tolerance of a clay mask, enriched with cellulobeads, kaolin, bentonite, thermal spring water, and vitamin B5 (panthenol), addressing concerns related to acne and oily skin, while promoting skin hydration and overall texture improvement. Various instrumental methods, clinical assessment by a blinded dermatologist and self‐assessment were used to evaluate the skin response to the clay mask.

It was found that the number of both open and closed comedones, sebum content, skin evenness, stratum corneum water content, and transepidermal water loss were all significantly improved, with changes noticeable from the immediate post‐treatment period and sustained throughout the course of the study. These results suggested that the studied clay mask is effective in mitigating prominent skin issues such as acne, hyperseborrhea, and skin dehydration, indicating immediate skin barrier enhancement. Various clinical trials have demonstrated the efficacy of commonly used clay minerals, including kaolin and bentonite for oily skin management, supporting the current results.^10^, ^11^, ^12^ Kaolin and bentonite are hydrated Aluminum Silicate and Aluminum Magnesium Silicate, respectively. They have the ability to absorb oil due to their large surface area, porosity, ionic charge, which make them effective ingredients in products designed to control oiliness.^13^ In addition, the studied clay mask contained cellulobeads. The hydrophilic microspheres possess a high capacity for moisture absorption, which can aid in retaining the skin's moisture balance, a characteristic that contributed to the significant increase in stratum corneum water content observed throughout the study.^14^ Furthermore, their small size (10 μm) and high bulk density (11.6 g/in^3^) suggest that they could provide a dense, uniform coverage on the skin surface, absorbing extra oil and potentially enhancing the mask's adherence and performance.

The alteration of sebum composition by the oil‐absorbing ingredients present in the clay mask plays a critical role in managing acne development.^15^ The mask's key constituents, kaolin and bentonite, can effectively extract surplus oil from the skin, thereby attenuating sebum production and decreasing the likelihood of pore blockage. Additionally, the cellulobeads help regulate the skin's moisture balance, mitigating any potential drying effects of the clays and preventing a compensatory overproduction of sebum. Thermal spring water, another ingredient, offers soothing and anti‐inflammatory benefits, which could assist in alleviating acne‐associated inflammation and promoting skin healing.^16^ Furthermore, the mask incorporates 5% vitamin B5 (panthenol), known for its role in skin soothing and anti‐inflammatory properties, could potentially contribute to the acne‐reducing effects of the product.^17^ Combined, they soothe the skin by decreasing interleukin (IL)−1α, which is an inflammatory factor highly expressed in comedones.^18^

Throughout the study, both objective and subjective assessments consistently indicated the safety of the clay mask. The significant decrease in skin dryness and total irritation score, along with reductions in sensations of itching and tightness from immediate post‐treatment to week 4, reflect the product's tolerability. These results, coupled with the observed improvements in skin condition such as decrease in TEWL, suggest that the clay mask is safe and suitable.

The self‐assessment results further reinforced the efficacy and acceptability of the clay mask. The majority of participants reported ease of application and wash‐off immediately after product use, highlighting the practicality of the product. Remarkably, after a week of use, all participants perceived the product's oil control effectiveness, suggesting a tangible, positive impact on skin oiliness. Furthermore, by the end of the study, over 95% of participants reported noticeable improvements in skin appearance, reduced visibility of skin flaws, and diminished skin discomfort.

In contrast to a previous clay mask study,^10^ which focused on acute effects over a short duration of 2 h, the present study extended the evaluation period to 4 weeks. This longer timeframe provides a more comprehensive understanding of clay mask's sustained benefits and potential effects on skin health. The extended duration not only captures immediate post‐treatment results but also offers insights into the cumulative benefits of regular usage.

While our study provides promising evidence for the efficacy of the clay mask, some limitations should be acknowledged. The lack of a significant decrease in porphyrin levels despite the reduction in oil levels is a notable observation from our study. One possible explanation could be that the skin microbiota, which includes the porphyrin‐producing bacteria (Propionibacterium), is resilient and can survive for some time even after oil levels decrease. Alternatively, the clay mask may have some effects on the skin microbiota that influence porphyrin production, as shown in a previous study that C. acnes might gain nutrition contained in the mask, such as glycerol, free fatty acids, especially in sebum‐deprived conditions, thus increasing the porphyrin.^19^ Further studies are needed to explore these possibilities and understand the complex interplay between skincare products, skin oil levels, and the skin microbiota.

In conclusion, this study provides compelling evidence for the efficacy and safety of the clay mask in managing skin conditions related to acne and oiliness, while promoting skin hydration and texture improvement. The clay mask demonstrated significant improvements in key skin parameters including the reduction of both open and closed comedones, sebum content, and transepidermal water loss, alongside enhancements in skin evenness and stratum corneum water content. Importantly, these improvements were observed from the immediate post‐treatment period and were sustained throughout the study. Furthermore, both objective and subjective tolerance assessments indicated high safety and acceptability of the clay mask, suggesting its suitability.

## CONFLICT OF INTEREST STATEMENT

No conflict of interest was reported by the authors.

## Data Availability

No.
